# Living Place Matters: The Duplicity of Shared Housing in the Young Adults of South Korea

**DOI:** 10.3389/fpsyg.2022.634905

**Published:** 2022-02-28

**Authors:** Nahyang Byun, Donghwa Shon

**Affiliations:** Department of Architecture, Chungbuk National University, Cheongju, South Korea

**Keywords:** shared house, co-living, living place, lifestyle, young generation, place-attachment

## Abstract

This study focus on the phenomenon of the preference for co-living among young adults that has manifested in South Korea. The study examines life in a shared house as a living place, which is the representative form of co-living that the younger adults in South Korea have been choosing. The objective of the study is to examine shared housing as living place matters and their possibility of being a home and house for the young generation. The study procedures included reviewing place attachment theory, analyzing the operational structure of shared houses, and interviewing residents to discuss the place attachment of the residential environment in shared houses. The young adult generation who chose to share a house display indecision on the issue of residential choices and behavior in terms of spatial possession. The results are as follows. Although co-living is a realistic residential choice for the reduction of residential costs, the majority of young adults experientially highlight the values of co-living rather than acknowledge the real reasons behind their choices. Such results signify that they recognize such limited residential choices as a means of temporary residence, not rooted to a living place, rather than an ordinal difference between the best and the second best, and ultimately the need to further consider the issues of continuous life and lifestyle on the foundation of the perspective of the universal life cycle of the young adult generation.

## Introduction

### Background and Objective

Choosing a house in accordance with the stages of one's life cycle is the physical foundation for quality of life. A house is a living place through which residents are rooted to their everyday life. Although young adults, in the early stages of their life cycles, are in a process of fully settling in as members of society, they are at a stage of economic vulnerability with limited housing choices (Doling, 1976; Kendig, 1984; Andersen, 2011). After young adults begin to live independently from the home that they lived in with their parents, it is widely accepted by society that they seek a temporary living place instead of owning a home, which many wait until marriage to do (Maalsen, 2018). In addition, the economically and residentially disadvantaged have found shared houses to be alternative living places. However, the meaning of shared houses is changing in the twenty-first century (Hemmens and Hoch, 1996; Xu et al., 2015; Holton, 2016; Maalsen, 2018). There are many shared house arrangements around the world, such as WeLive in the United States, where start-up entrepreneurs co-work and shared living quarters, and Old Oak in the UK where various room services and ancillary facilities are available for tenants (Kim, 2015; Woo et al., 2019; Bergan et al., 2020; Kim et al., 2020). These are cases of private rental housing that secure the convenience of transportation and living space in metropolitan areas where the burden of housing costs is high. Since the 2000s, private rental housing has spread mainly among young adults (Hirayama and Ronald, 2007; Holton, 2016; Druta and Ronald, 2020; Kim et al., 2020). There is still controversy over whether a shared house is a reasonable or inevitable option as a housing choice (Kenyon, 1999; Kenyon and Heath, 2001). Because of different social, physical, and temporal interactions such as conflicts between residents and privacy issues around shared houses, it is still too early to judge if they can function as a long-term sustainable living place (Clark et al., 2017, 2019; Orlek, 2017; Nasreen and Ruming, 2020).

Economic instability and a poor residential environment for young adults are significant social issues in South Korea. Korea has experienced compressed modernization in Asia (Park, 2015, 2016; Byun et al., 2018). Until the present, the supply of public rental housing was based on households with married couples and households of those supporting elderly parents with residential vouchers offered to people with lower-incomes. This is the result of a long-held social perception that single young adults correspond only to the stage of living away from their parents prior to the formation of their own family in their life cycles due to studying at a university and finding a job (Kang, 2017).

The issue of housing for single-person households in the young adult generation has become a prevalent social problem. Together with the rise in the jobless population and delayed social entry, it takes a long time to find a job and economic independence is delayed. There has been no clear housing policy to support young adults who do not belong to a defined social class. A policy vacuum for young adults who do not qualify for any class places them in economic instability and a poor residential environment. The majority of shared houses are operated within a sublease framework, which creates a legal blind spot where the residential rights of tenants are not guaranteed. There is a lack of regulatory policies on house sharing, minimum hygiene conditions, and limitations on people sharing the same residence (Byun et al., 2018). In addition, the legislation is not transparent enough to regulate the legal responsibility and accountability of tenants, homeowners, and managers (Byun, 2020). For a long time, Korea's social perception that young adults were occupants of temporary households rather than independent households had an impact on housing policy and the housing market as well as housing choices. Although a stable residential environment must be secured, in-depth consideration of the housing of young adults has not been considered.

This study focuses on the phenomenon of shared house preferences of young adults in Korea. This research begins with living “place” matters and raises two research questions: will shared houses be settled living places? Can shared houses offer new place values in terms of living place based on the concept of place attachment?

The objective of the study is to examine shared houses as living places and the possibility of them being a home for the young adult generation. The study procedures include reviewing place attachment theory, analyzing the operational structure of shared houses, and interviewing residents to discuss the place attachment of shared houses as residential environments.

### Place Attachment Theory

It is necessary to discuss the meaning of the environment at a spatial level and to consider person- and place-centered approaches. In particular, the features of the built environment are identified through the process of exploring the relationship between “place” and “person.” The concept of place attachment is applied in various ways, from the relationship of a specific place and person (Hidalgo and Hernandez, 2001; Giuliani, 2003) to the “sense of place” that a person needs (Relph, 1976). In the study of housing, place attachment is the basis of the theoretical framework to understand the relationship between the behavior of residents and the residential environment (Raymond et al., 2010; Fornara et al., 2019). The place attachment of residents to their living place plays a significant role in helping them feel that they belong in a residential environment and neighborhood in a community (Kamalipour et al., 2012).

Scannell and Gifford (2010a) defined a “tripartite model of place attachment” as organized person-place-process dimensions. This mode has meaning as a general definition of place attachment, which comprehensively presents a common and consistent concept in the discussions of researchers on place attachments. This study is based on the place attachment theory of the Scannell and Gifford tripartite model to examine shared houses as living places.

First, in the model of place attachment, the person dimension can be explained at the individual level and group level (Scannell and Gifford, 2010a). The individual level involves experiences, realizations, and milestones, and the group level is close to meanings of genders, cultures, and religions (Scannell and Gifford, 2010a). In a range of living places, shared houses involve both the individual level and group level as personal connections and spatial sharing among members.

Second, the process dimension of place attachment is psychological for affect, cognition, and behavior (Scannell and Gifford, 2010a). Affect is an “emotional” meaning-happiness, pride, and love- involved in person-place bonding (Manzo, 2003). Cognition contains memory, knowledge, schemas, and meanings. The behavior level is an expression of actions by proximity, maintaining, or reconstructions of place. These three concepts are separate terms. However, it is necessary to consider the concepts comprehensively to review the process dimension of place attachment. In the living place, issues of residents' satisfaction with environments, relationships between residents (flatmates), and the meaning of living together in shared houses should be discussed.

The place dimension encompasses perception of “the place itself,” but also measures the social and physical dimensions of attachment (Hidalgo and Hernandez, 2001; Scannell and Gifford, 2010a,b). Hidalgo and Hernandez (2001) suggest that community attachment was identified with social bonding and physical rootedness. Shared-house residents have a similar living range of spatial level from the house (or room) to the community area. This means that the physical place overlaps or is the same, but the social place can be different. Furthermore, the length of residence and plans to stay can be predicted with physical attachment or not “rootedness” in a living place (Riger and Lavrakas, 1981). These dimensions are highly related to the planning and design of the residential environment by considering sharable boundaries.

## Materials and Methods

### Online Data Collection

We collected raw leasing data from online platforms and materials from shared house organizations that manage many units in metropolitan areas (Come&Stay, 2019). The first form of data was collected from related websites, and we further interviewed the representatives or managers of shared house organizations to obtain more detailed information. According to data announced by an online platform specializing in shared housing in Korea, based on the number of rented households, this began with four cases in the second half of 2012, which rapidly increased to 329 cases in the fourth quarter of 2017. There were 2,407 beds from 1,398 rooms rented, indicating that the majority were for two occupants in each room (Come&Stay, 2017). The principle that operates shared housing in Korea is an organization that specializes in the operation and management of shared housing. Although the status of the industry has not been accurately and specifically discerned, there is a trend toward an increase in the number of relevant operating organizations including limited companies, housing cooperatives, social corporations, and individuals (rental business operators). The activities of such operating organizations can be categorized into residential land purchasing, planning, design, construction, and maintenance, with cases in which the organization specializes in the consigned management of shared housing. From the data collected from online platforms, we confirmed that many tenants, with varying lease durations, were sharing a house (Table 1).

**Table 1:** Management status of shared housing.

**Components**			**Avg**.	**Min**.	**Max**.
Housing cost (1,000 KRW)	Monthly rent	Lowest	360	110	570
		Highest	470	190	850
		Deposit Lowest	1,100	200	10,000
		Highest	1,500	200	50,000
	Utility/maintenance cost		47	10	120
Living condition	Residents' age	Youngest	19.8	18	22
		Oldest	35.9	50	50
	Minimum contract length (months)		4.6	0.5	12
	Number of residents (people)		7.5	1	48
Housing area (m^2^)	Total area		116.3	38.0	565.0
	Area per person	Min.	9.9	4.0	38.0
		Max.	17.7	6.6	67.0
Dwelling space	Number of bedrooms		4.3	1	37
	Number of toilets/shower booths		2.3	1	24

*Data were collected from 1 December to 31 December, 2017*.

### Focus Group of Residents

The purpose of the focus group was to understand the livelihood of shared house residents based on user experience by identifying their current residence and their perception of that residence. We also tried to understand the social sharing of daily lives and interactions between residents in addition to the physical sharing of a place. Interviews were carried out with 26 residents, comprising five focus groups of the shared houses in a metropolitan city of Korea—five residents of “ABLE HOUSE” on February 2, 2018, in Seoul (SO), five residents of “Gumgultong” on January 10, 2018, in Daejeon (DJ), 5 residents of “BUNKERHOUSE” on February 12, 2018, in Daegu (DG), five residents of “Gongmyung” on January 26, 2018, in Gwangju (GJ), and 6 residents of “SONG's VILL” on December 23, 2017, in Busan (BS). The cases of the shared houses in which the subjects of the interview lived represent the brand names of various branches in each of the regions. After creating respondent-specific and semi-structured questionnaires for five groups in each city, the author interviewed the residents regarding their living situations.

The focus groups were composed of three parts where discussion of current living, perception of shared houses, and social and physical sharing was based on place attachment concepts (Table 2). In the first part, the interviewer asked whether the room type is a shared room. On the satisfaction with current living, the residents evaluated each content of residential environment factors on a 5-point Likert-type scale (from 1 = not at all to 5 = completely). For discussion, the residents responded to the questions, and then talked freely about the questions related to issues or expressed their thoughts in no particular order.

**Table 2:** Focus group.

**Method**	**Items**	**Interview questions**	**Place attachment concepts**		
			**Person**	**Place**	**Process**
Semi-structured questionnaire	Current living	House information channels	–	–	–
		Room type (share or not)	–	v	–
		Length of residence / Plans to stay	–	v	–
		Past living experience of shared housing	v	–	–
		Advantages and disadvantages	v	v	v
		Satisfaction of residential environment.	v	v	v
Topic discussion	Perception of shared house	Appropriate length of stay	–	v	–
		Sharable scope	v	v	–
		Priorities in selecting a house	v	–	v
		Importance for life in a shared house	–	–	v
		Acquaintance and family perception	–	–	v
	Social and physical sharing	Activities or tasks shared among residents	v	–	v
		Current usage of common spaces	–	v	v
		Acceptance of other residents	v	v	–
		Willingness to live in other regions	v	V	v

The focus group was conducted as follows. In the preparatory stage, the status of shared house residents was identified with a written survey of operators of each sharehouse. Prospective interviewees were recruited and participated in interviews with the author. Interviews were conducted in five sessions for each interviewee, with each session lasting about 130 min. Interviews consisted of a warm-up interview and a subject interview. For the warm-up, a semi-structured questionnaire was distributed by the interviewer. The residents first replied to each question and then confirmed their answers in an interview. For the subject interviews, the interviewees freely answered the questions. A research assistant typed details of the interviews during the sessions with voice recordings.

## Results

The five shared house organizations, marketed and recruited university students through online platforms, social media, and their websites from 2014 to 2017. They offer a wide range of unique tenant management and lifestyle services such as events and social meetings. The five organizations operate other businesses in addition to the shared houses. Able House manages 19 shared houses for exchange students and university students, develops computer software and applications, and manages other real estate properties. Gumgultong began to manage share houses by participating in local projects with government grants. Gongmyung focuses on residence building leasing services. Bunkerhouse operates residence databases in the local area and house makeovers. Song's Vill began its business as a shared house provider and has now expanded to room sharing management.

A summary of resident characteristics shows that 10 residents were male and 16 were female (Table 3). Their average age was 26 years, with those in their late 20s accounting for more than half the residents. In total, 67% (18) were college students (11) or those preparing to find a job (7), and all were unmarried. All residents of DG were university students and females. SO were university students, both male and female. Residents of BS were acquaintances. Residents of GJ and BS were students and workers. DG, SO, and BS were in a similar living radius. GJ and BS were not in a similar living radius because they lived away from workplaces and universities. Although the residents' hometowns were different and they currently live together in the same shared houses, each group has a similar spatial range to live in. There is a possibility of place attachment based on a sense of community.

**Table 3:** Summary of respondents.

**Components**	***N* (%)**
Gender	
Male	10 (38.5)
Female	16 (61.5)
Age	
20–24	9 (34.6)
25–30	14 (53.9)
31–34	3 (11.5)
Education	
High school or less	4 (15.4)
2-year university	11 (42.3)
4-year university	10 (38.5)
Graduate school	1 (3.8)
Job	
Employed	8 (30.7)
Not employed (student)	11 (42.4)
Not employed (looking for a job)	7 (26.9)
Average monthly income (10,000 KRW)	
100	10 (38.5)
100–200	9 (34.6)
200–	3 (11.5)
None	4 (15.4)
Average monthly living cost (10,000 KRW)	
50	10 (38.5)
50–100	12 (46.1)
100–	4 (15.4)

### Current Living

To summarize the results for current shared house living conditions, the respondents were mostly satisfied with living in shared houses. They stressed that there were no problems with sharing dwelling spaces such as living rooms, kitchens, and bathrooms because they decided to live in a shared house to save housing expenses. Online platforms were the central source for exchanging information and finalizing contracts, and the shared house management and tenants communicated and negotiated through social media.

Half of the respondents reported their channel for obtaining information on shared houses online (Internet search, blogs, and social media promotion), while 46% were referenced and introduced by acquaintances. For room type, 42% of respondents had single-occupancy rooms, 27% had double-occupancy rooms, and 23% had rooms for three or four persons. Twenty-four residents answered negative when asked whether they had previously resided in shared houses.

Most of the residents did not live in shared houses for a long period, and it was unclear whether some would continue to live in the same shared house. Of the respondents, 77% had lived <1 year in their shared houses, while 46% answered “don't know” regarding their future length of stay. This shows that the lengths of time of residence are not long and plans to stay in shared houses are not made based on a long period or are unclear. Considering that most of the residents rent rooms by the month in shared houses, these results mean that physical attachment is not strong or “rootedness” is weak at the home level.

For the pros and cons of shared houses, the largest portion of respondents cited “reduced housing cost” as an advantage and “common spaces” as a disadvantage. Next to reduced housing cost, shared house residents chose “sense of security and sense of belonging” as a further advantage. Sense of security and sense of belonging are related to social and physical attachment based on the living place. The functions of place attachments include security, which is relative when compared to living alone. Shared houses provide for more than one person, and a common physical environment such as a living room and kitchen for residents to use together. Therefore, residents feel like they belong in the shared house.

Housing costs were low, comprising 70–80% of the monthly rent, which varied with the number of bedroom occupants. However, the deposit was double the monthly rent. Maintenance fees, which included electricity, gas, and water charges, were either split among the residents or paid by the shared house operating body.

In terms of their satisfaction with the residential environment, the residents were very satisfied in every way. Most items in the residential environment were rated over the “satisfied (4)” level. It is necessary to attach to the place the appropriate level of satisfaction in the residential environment. In this context, the residents saw their shared house as a living place. The interpretation of this outcome needs to take into consideration the fact that respondents had a pre-existing positive opinion because they had chosen to share houses instead of the other types of residences and had agreed to the interview. Nevertheless, the desire to use shared houses for a short period reflects the negative side for shared houses to become deeply established with place attachment.

### Perception

#### Appropriate Length to Stay and Sharable Scope of Share Houses

The results of appropriate lengths of time to stay in shared houses are as follows. Most respondents answered that it is appropriate to live in shared houses for a short period rather than a long period. Thirty-six percent responded under 1 year, 34% under 2 years, 19% more than 3 years or as needed, and 11% (3 persons) answered: “I don't want to live in share houses.” The results mean that residents regarded shared houses as short-term living places. That is assuming they moved in the next few years. Studies need to look at the results of the length of residency and plans to stay in the current living part. The sense of community contains a connected lifestyle or interest, but it is not clear if this will go on for a long time.

In shared houses, the physical sharing level such as rooms, bathrooms, kitchens, and living rooms, and the social sharing levels such as socializing were both quite high among the residents. For their perception of shared houses and their life in shared houses, 42% responded “OK except for the bedroom” and 34% responded “OK except for the bed,” referring to the sharable spaces and facilities, thus exhibiting the same results as the current type of residence in shared houses. Over half of the respondents were currently in a shared room, which matched the results of the room type questions. Regarding sharable facilities (such as furniture and electronics), a small number of respondents selected furniture such as storage closets and desks, while 50% answered that they could share food items, personal items such as computers, and other items according to personal tastes. Other specific items all registered high ratios. This differs from the outcome on “willingness to share spaces and facilities” in the survey of the young adults' current residence and demand for housing.

#### Selecting Houses and Perceptions of Others

The location and rent of shared houses are important considerations when selecting houses. The priorities are location, program by management, room type, and rental cost. When choosing a house (including a shared house), location and rent are key factors. Here, it is meaningful that location and rent fee come first rather than room type and others. The results showed that shared houses were set around a community of place because geographical location means a place dimension of attachment that overlaps community boundaries physically and socially.

The perceptions of the residents' parents or acquaintances were positive. Some parents of residents thought that living in shared houses was safer than living alone from a security aspect. Some residents' acquaintances worried about living in shared houses. They lacked information on shared houses and had no experience living in them. However, they thought that the experience of living in shared houses might come in handy 1 day.

### Social and Physical Sharing

Social and physical sharing in shared houses involves three dimensions of place attachment: person, place, and process. The degree of social sharing among residents demonstrated the following: residents attended resident meetings as well as regular gatherings or meetings supervised by the shared house operators, and residents discussed regulations and provide suggestions. Sometimes, team leaders selected for different shared households served as points of contact for the operating body manager.

#### Experience Activities Among the Residents

Activities and episodes can consider the person and process dimensions. It could be shown that a community among residents was formed. For example, episodes of congratulating co-residents for birthdays or taking language or music lessons together were often mentioned. House members went beyond mere exchanges of greetings among members and joined in collective life.

*BS_A: When I was ill, a roommate offered me medication and accompanied me to the hospital. On birthdays, I have a party and I drink together with other residents on days when I feel particularly severe stress*.

*PS_B: I learned to ride a bicycle from my roommate*.

*GJ_E: It is great to live in a shared house since I do not become lonely and can share fried chicken whenever I want to eat it. House cleaning can be stressful, but can be markedly lessened by doing major cleaning together on a set date along with segregated disposal of waste and garbage*.

*DG_A: All five people started sharing the house together at the same time, and we all do weight training and kick-boxing together*.

*SO_D: I offered free Korean lessons and help in his major area of study to a French house mate*.

At the individual level of the person dimension, a resident has experience with other residents during their daily life through learning something from each other. The interview results showed both “community of interest” to connect lifestyle and “community of place” in the same house. Two community types are based on the networking among the residents. This means that residents agreed to the meaning of being together and interpersonal behavior. Thus, being together and interpersonal behavior are strongly involved in the three dimensions of place attachment.

#### Current Use of Common Spaces

The current use of common spaces partially shows the physical sharing level and residents' living behavior in shared houses. Residents expressed differences in how often they used and how they behaved in the living room. Some residents rarely used the living room, while others frequently used the living room for eating dinner or watching TV. This shows that social sharing, such as socializing and bonding activities between tenants, is also correlated with the physical sharing of the space.

Residents of BS, SO, and DG shared houses were open to using a common living room, whereas DJ and GJ residents did not prefer to use a living room. As to why a living room is not preferred, three DJ residents pointed out that there was a problem with air conditioning and heating in the living room. Two residents of GJ shared houses used the living room to dry their laundry and said that it becomes loud when non-resident friends visit. Such a difference in preferences may be explained by the types of shared house residents. DJ shared houses were occupied by young office workers and college students. GJ shared houses were occupied by college graduates seeking jobs and college students, and residents of other shared houses were college students. The residents who are agreeable to sharing a common living room were students attending the same college (their majors may differ), or students attending different colleges, which were located at a close distance, and whose students move in a close circle.

*SO_D: We frequently have dinner together in the living room*.

*BS _D: We all use the living room together*.

*DJ_B: During the summer, we get together in the living room more often and for longer periods of time. I chat with the others a lot since I do a lot of work in the kitchen because it has a desk which I can work on*.

*GJ_D: I am not able to use the living room as much as I want to since we hang laundry there on racks*.

*DG_E: I tend to stay in the living room for quite a long time since I watch TV, take naps, eat meals and play games there*.

#### Acceptable Residents

For the acceptance of other shared houses residents, the standard presented most frequently was “age.” Normally, generational differences could have an effect in many ways. The residents anticipated problems including limitations in communication and establishment of consensus due to differences in value systems if the age difference among occupants was too large. The results also mean that shared house residents want to live with similar age groups because, with the same generation peer group, the cultural and social background could be understood. The results showed that there are possibilities of place attachment at the group level in terms of personal dimensions.

There were no restrictions on foreigners. However, the problem of providing security for occupants in the case of the presence of mixed genders was highlighted by female respondents. The propensities of occupants were also mentioned from the perspective that there could be inconveniences in sharing opinions or considering others while living together.

*BS _A: There is a need to restrict the age of occupants because there could be problems in communication if the ages differ significantly*.

*GJ_A: Age-group peers would feel more comfortable living together*.

*SO_C: There must be a limited range of occupant ages. It would be more possible to establish a consensus if the occupants are neither too young nor too old*.

*DG_D: Difference in age is important*.

#### Willingness to Live in Other Regions

In terms of their willingness to live in shared houses in regions other than their current location after they graduate from college, the respondents' answers were varied. Some respondents wanted to live in a standard house instead of a shared house if they were able to pay for their housing by finding a job after college graduation. They also said that they would positively consider a shared house for corporate employees if public transportation were convenient, depending on the shared house operator, residents, and housing costs.

*DJ_A: I will be living in a sharehouse only until I save sufficient money for a security deposit to rent a single-room residence*.

*DJ_C: I will live in a sharehouse if it is close to my workplace and then move to my own residence once I save sufficient funds to cover the security deposit*.

*SO_A: I wish to live in a sharehouse for working people only. I want to live in a shared house or keep co-working even after I am employed*.

*SO_C: Once I am employed, I think shared housing may not be appropriate due to the need for dating and marriage*.

The previous interview results showed that residents considered shared houses for short-term living. In the same vein, the results of willingness to live in other regions also showed that residents were subject to move their living place, but not to shared houses. What is interesting is that the results show an ambivalent attitude by residents. Other results of social and physical sharing showed that it is based on place attachment in part. However, the results of willingness to live in other regions weakly showed the concept of attachment in person-place bonding.

## Discussion

The following implications could be derived from the results of interviews with the current residents of shared houses. First, there is hardly a tendency of rootedness in the current living situation in shared houses. The study results illustrated the characteristics of short-term residences in shared houses due to indefinite periods of residence in the future and signifies that temporary residence is prevalent rather than stable and continuous residence in shared houses. The main reason for living in a shared house was to save on the cost of residence, and subjects mostly wanted to live there for the short-term of <1 year. Current residents chose a shared house because the costs were lower than the market value of separate residences in the areas around universities.

Second, the person and place dimensions of place attachment showed strong social and physical sharing aspects. Those in their 20s accounted for the largest proportion of current residents with high levels of satisfaction and associated living in a shared house with the establishment of consensus among their peer group. If the housemates were students from the same university, they displayed a high level of understanding of each other's lives since their academic curriculum and schedules were similar. It was stronger in the case of DG, where female students lived. These could be collective cultural characteristics of Korean society when compared to shared houses in other countries. In the case of exchange students, the operator of the shared house provided various amounts of information or linguistic support to aid their daily convenience. Where residents were working people, they highlighted the sense of belonging and stability through interactions with other residents after working hours, in comparison to living alone, as constituting the foremost advantage.

Third, place attachment concepts of shared houses showed conflicting results. Shared houses were considered an option for a residential format depending on specific situations. The results of the interviews illustrate that living in shared houses cannot be seen as simply an issue of choosing between living alone or with others. This is because the residents who consented to the interviews displayed conflicting attitudes about choosing to live in shared housing due to logistical issues such as high residential costs while having to endure the inconvenience and privacy issues associated with having to share living spaces with others. The conflicting attitude of residents showed a duality between the housing demands of young adults and real life. Moreover, despite the high level of satisfaction in the residential environment, the tendency of wishing to reside only for a short period of <1 year illustrates that shared housing needs to be approached in greater depth as a temporary residence and with consideration of resources available in the vicinity of the house (Druta and Ronald, 2020). This tendency is the most important implication to consider when approaching the concept of shared housing as an alternative residential model (Kim et al., 2020). This is because Korean house shares are at a crossroads on whether they will stay temporary residences or become homes, which is the principal difference from cases abroad. As a result, shared houses fundamentally provide physical attachment features, but are limited to developing place attachment like the home level today.

Those of the young adult generation who chose shared residences display indecision on the issue of residential choices and behavior in terms of spatial possession. The concept of place attachment was shown strongly in residents in relation to aspects of shared house living. However, some parts were shown slightly. These findings are an important basis for whether a shared house could be a settled living place. To be a settled living place, it is necessary to improve the residents' place attachment close to the level of a home. However, it is possible to be a settled living place for young adults even with the double meaning of residents. Shared houses include physical features such as the place itself and place as spatial level. To develop the place attachment of the residential environment, the values of living place could be expanded more. For the results of the focus group, although it was a realistic residential choice for the reduction of residential costs, the majority of young adults are experientially highlighting the values of “co-living” rather than acknowledging the real reasons behind their choices. This is illustrated in their behavior and spatial uses based on the process and place dimensions of place attachment. The tenants emphasized the advantages of being able to use spaces other than individual bedrooms by sharing the residential costs for the common areas, and the behavior in occupying the common areas such as the kitchen appeared to have a wide variance depending on the extent of fellowship among the shared house tenants.

The duplicity of the shared house is based on the residents' conflicting data of high satisfaction level, but they did not live in a shared house long-term. This shows a cognitive dissonance and inconsistency in their attitude and behavior. The biggest reason to choose house sharing is to save on the costs, but they justified the reasons by expressing other positive points. The purpose of choosing a shared house to reduce housing costs and the value of living within a group are not always compatible. The co-living of the start-up class in other countries is consistent with the purpose of building and promoting human networks through collaboration and living values (Clark et al., 2019; Bergan et al., 2020), because of residents who are in the one-person band or professions. Korea's shared houses indicate that for some issues, co-living value, and culture can be discussed in a similar vein as other countries. However, the issues of sustainable living places and being a home need to be discussed in a different context. The increased use of shared houses has not been happening for long in Korea, and there are only a few cases of long-term residence, which is a limitation for the insufficient narrative approach (Nasreen and Ruming, 2020).

## Conclusion

For shared housing to be considered an alternative form of housing, there is a need to ponder the possibility of expansion of the prospective consumer base for such residences (Holton, 2016; Maalsen, 2018). This is because shared housing is currently in the transition stage, with inadequate preparation of foundations within the institutionalized system and a lack of social awareness despite the demand, mostly among those in their 20s. Therefore, Korean society is at a crossroads whereby shared housing continues to be considered only as a place of temporary residence for those in their 20s, rather than performing the function of providing more sustainable and stable homes. The possibility of shared housing functioning as an alternative form of housing does exist since the young adult generation is opting to save on economic costs and gain the social value of living together with others while residing practically in shared housing for short periods of time rather than living alone. However, shared housing is still seen as being limited to temporary residences. Adults in their 20s use it to reduce residential costs given their economic vulnerability at this point in their life cycle. At the same time, shared housing has the potential to become an alternative form of housing for a wider range of age brackets, given the increase in the number of one-person households throughout the entire demographic range of Koreans.

This study has the following limitations. First, the sample size of five focus groups of 26 residents is small. Second, this study deals with urban shared homes and not rural shared homes since the research set bounds to major city regions. Third, it is common to be faced with a housing problem in a metropolitan area for young adults from Europe, America, Asia, and other regions, and young adult generational living is also different in terms of economic and socio-cultural aspects. However, the research results are meaningful and illustrate the that living place of young adults matters. Fourth, the results signify that young adults are recognizing limited residential choices as a means of temporary residence rather than an ordinal difference between the best and the second-best, and ultimately implying the need to further consider the issues of sustainable living places and lifestyle for the foundation of the perspective of the universal life cycle of the young adult generation.

## Data Availability Statement

All datasets generated for this study are included in the article/supplementary material, further inquiries can be directed to the corresponding author/s.

## Ethics Statement

Ethical review and approval was not required for the study on human participants in accordance with the local legislation and institutional requirements. The patients/participants provided their written informed consent to participate in this study.

## Author Contributions

NB carried out the research in general and wrote the manuscript with support from DS. DS investigated parts of the literature review and developed the discussion parts. Both authors discussed the results and contributed to the final manuscript. Both authors contributed to the article and approved the submitted version.

## Conflict of Interest

The authors declare that the research was conducted in the absence of any commercial or financial relationships that could be construed as a potential conflict of interest.

## Publisher's Note

All claims expressed in this article are solely those of the authors and do not necessarily represent those of their affiliated organizations, or those of the publisher, the editors and the reviewers. Any product that may be evaluated in this article, or claim that may be made by its manufacturer, is not guaranteed or endorsed by the publisher.
